# Epithelial-derived galectin-9 containing exosomes contribute to the immunomodulatory effects promoted by 2’-fucosyllactose and short-chain galacto- and long-chain fructo-oligosaccharides

**DOI:** 10.3389/fimmu.2022.1026031

**Published:** 2022-12-22

**Authors:** Veronica Ayechu-Muruzabal, Merel de Boer, Bart Blokhuis, Alinda J. Berends, Johan Garssen, Aletta D. Kraneveld, Belinda van’t Land, Linette E. M. Willemsen

**Affiliations:** ^1^ Division of Pharmacology, Utrecht Institute for Pharmaceutical Sciences (UIPS), Utrecht University, Utrecht, Netherlands; ^2^ Danone Nutricia Research, Utrecht, Netherlands; ^3^ Center for Translational Immunology, The Wilhelmina Children’s Hospital, University Medical Center Utrecht, Utrecht, Netherlands

**Keywords:** galectins, exosome, non-digestible oligosaccharide, intestinal-epithelial cells, mucosal immunity

## Abstract

**Introduction:**

Early life exposure to non-digestible oligosaccharides (NDO) or microbial components is known to affect immune development. NDO in combination with a TLR9 agonist mimicking bacterial triggers (CpG) promoted the secretion of galectins through unknown pathways. We aimed to study the contribution of exosomes in epithelial galectin secretion and subsequent immunoregulation upon exposure to a mixture of NDO by inhibiting exosome biogenesis.

**Methods:**

Human intestinal epithelial cells (IEC) (FHs 74 Int or HT-29) were apically exposed to 2’-fucosyllactose (2’FL) and short-chain galacto- and long-chain fructo-oligosaccharides (GF), alone or with CpG. Basolaterally, non-activated or αCD3/CD28-activated peripheral blood mononuclear cells (PBMC) were added. After 24 h incubation, IEC were washed and incubated in fresh medium to analyze epithelial-derived galectin secretion. Additionally, before exposure to NDO and CpG, IEC were exposed to GW4869 to inhibit exosome biogenesis. After 24 h of incubation, IEC were washed and incubated for additional 24 h in the presence of GW4869, after which epithelial-derived galectin secretion was studied. Also, epithelial-derived exosomes were isolated to study the presence of galectins within the exosomes.

**Results:**

Compared to CpG alone, exposure to 2’FL/GF mixture and CpG, significantly enhanced Th1-type IFNγ, and regulatory IEC-derived galectin-9 secretion in the HT-29/PBMC model. Similarly, in the FHs 74 Int/PBMC co-culture, 2’FL/GF induced immunomodulatory effects in the absence of CpG. Interestingly, galectin-9 and -4 were present in CD63-expressing exosomes isolated from HT-29 supernatants after IEC/PBMC co-culture. Exposure to GW4869 suppressed 2’FL/GF and CpG induced epithelial-derived galectin-9 secretion, which subsequently prevented the rise in IL-10 and reduction in IL-13 secretion observed in the HT-29/PBMC co-culture model upon exposure to 2’FL/GF and CpG.

**Discussion:**

Exposure to 2’FL/GF and CpG or 2’FL/GF promoted Th1-type regulatory effects in HT-29/PBMC or FHs 74 Int/PBMC co-culture respectively, while Th2-type IL-13 was reduced in association with increased galectin-9 release. Galectin-9 and -4 were present in exosomes from HT-29 and the inhibition of exosome biogenesis inhibited epithelial-derived galectin secretion. This, also affected immunomodulatory effects in IEC/PBMC co-culture suggesting a key role of galectin expressing IEC-derived exosomes in the mucosal immune regulation induced by NDO.

## 1 Introduction

Non-digestible oligosaccharides (NDO) in human milk provide the infant with a unique source of energy, and support the growth and development of key organs and systems as well as contributing to the establishment of the microbiome. Specific NDO derived from milk or plant sources such as a 9:1 mixture of short-chain galacto- and long-chain fructo-oligosaccharides (GF) have been manufactured to mimic the amount and structure diversity of NDO in human milk. This NDO mixture was shown to promote the growth of beneficial commensal bacteria as well as supporting the immune system (1). Direct effects of these NDO were investigated in an *in vitro* co-culture model built to study the crosstalk between intestinal epithelial cells (IEC) and activated peripheral blood mononuclear cells (PBMC). In association with CpG oligodeoxynucleotides (ODN) or bacterial DNA from *Bifidobacterium breve* M-16V, the GF mixture was found to enhance regulatory type Th1 cytokines, while lowering Th2 type response, driving away from the allergic phenotype (2, 3). Galectin-9 was identified as the epithelial-derived factor responsible for shaping the phenotype of the immune response (2). Furthermore, in a murine model for cow’s milk or hen’s egg allergy, a reduction of allergic symptoms was observed upon combination of GF with *Bifidobacterium breve* M-16V. These effects were associated with increased galectin-9 levels in serum and intestine (4–6). Additionally, in children affected with atopic dermatitis fed hydrolyzed milk formula supplemented with GF and *Bifidobacterium breve* M-16V, serum galectin-9 levels increased while their symptoms reduced (5, 7). One year later, these children had a lower risk of developing asthma like symptoms and less asthma medication was prescribed compared to control group (8). In an *in vitro* IEC/PBMC co-culture model designed to study the crosstalk of IEC and immune cells, similar immunomodulatory effects were observed upon exposure to 2’-fucosyllactose (2’FL), one of the most abundant NDO in human milk, and GF when combined with CpG, mimicking a bacterial trigger (9). Both, 2’FL and GF in combination with CpG, promoted Th1-type regulatory immune effects. Epithelial-derived galectin-3, -4 and in particular galectin-9 were thought to be involved in promoting immunomodulation (9). Furthermore, the neutralization of galectin-9 using Tim3Fc fusion protein prevented the increase in IFNγ and IL-10 secretion in IEC/PBMC indicating the involvement of galectin-9 in the observed immunomodulatory effects (2).

Galectins are carbohydrate-binding proteins with diverse intracellular and extracellular physiological functions like supporting epithelial homeostasis and immune functions (10). Galectins are synthetized in the cytosol and lack a signal sequence thus, are thought to be released by non-classical secretion mechanisms (11). One of the non-conventional pathways by which galectins are thought to be secreted is *via* extracellular vesicles (11). Extracellular vesicles (EV) contain biomolecules (proteins, lipids and nucleic acids) which are relevant for intercellular communication that can modulate immune responses locally as well as systemically (12–16). Exosomes are small extracellular vesicles (30-200 nm) which are originated from multivesicular bodies and released into the extracellular compartment by fusion with the plasma membrane (12–16). There, they participate in cell-to-cell communication as well as contributing to transport, storage and release of proteins (12–16) that mediate intercellular communication but also contribute to maintain cellular homeostasis (17, 18). Many cell types including enterocytes and immune cells are known to secrete exosomes with diverse physiological functions (12, 15). In particular in the gut, EV secreted from IEC can interact with the immune cells present in the lamina propria and thereby control intestinal homeostasis (13). Previously, exosomes derived from T84 IEC were shown to interact with dendritic cells which in turn led to T-cell activation (19). Besides T84, also HT-29 IEC were shown to produce CD63-expressing exosomes (20). Furthermore, the secretion of exosome-containing galectins has been previously described (21, 22). Nevertheless, the exact mechanism regarding how galectins are released by IEC remains poorly understood.

Ceramide is an abundant component of the exosomal membrane which is produced upon hydrolysis of sphingomyelin and its accumulation is required for the formation of exosomes (22). Downregulation of the ceramide production by a neutral inhibitor of sphingomyelinase (nSMase) resulted in the inhibition of the production of exosomes (23). In this regard, the neutral sphingomyelinase inhibitor GW4869 has been used to potentially block the mechanisms required for the biogenesis and secretion of exosomes (24, 25).

IEC were shown to secrete epithelial-derived galectins upon exposure to NDO and CpG in an *in vitro* co-culture model used to study the crosstalk between HT-29 and immune cells (2, 3, 9, 26). Due to the carcinogenic background of the HT-29 cell line, we aimed to investigate if other non-carcinogenic epithelial cell lines could promote immunomodulatory effects upon exposure to NDO in the presence or absence of CpG. Hereby, we aimed to study whether NDO alone or in combination with CpG could promote immunomodulatory effects in a human fetal intestinal cell line (FHs 74 Int), similar to the effects shown for HT-29 before (2, 9). In addition, we studied if the inhibition of exosome biogenesis by GW4869 impaired the epithelial-derived galectin secretion that was promoted upon exposure of IEC to NDO and CpG. Moreover, we studied if the inhibition of galectin secretion interfered with the immunomodulatory effects observed in IEC/PBMC upon exposure to NDO and CpG, to contribute to the understanding of the roles of galectins and exosomes in immunomodulatory responses in the intestinal mucosa.

## 2 Material and methods

### 2.1 Intestinal epithelial cells

Human FHs 74 Int (ATCC, CCL-241™) and human HT-29 (ATCC, HTB-38™) cell lines were used as models for IEC. FHs 74 Int were grown in T25 flasks (Greiner Bio-One, Alphen aan den Rijn, The Netherlands) using Hybri-care medium (ATCC, 46-X) supplemented with 30 ng/mL epidermal growth factor (EGF), 10% fetal-calf serum (FCS), penicillin (100 U/mL) and streptomycin (100 µg/mL). HT-29 were grown in T75 flasks (Greiner Bio-One) using McCoy 5A medium (Gibco, Invitrogen, Carlsbad, CA, USA) supplemented with 10% FCS, penicillin (100 U/mL) and streptomycin (100 µg/mL). Medium was refreshed every 2-3 days and cultures were maintained at 37 °C and 5% CO_2_. One week before the experiments, IEC were seeded in 12-well transwell inserts (Costar Corning Incorporated, New York, NY, USA) by diluting 4 times for FHs 74 Int and 8-10 times for HT-29 based on surface area. Confluent IEC monolayers were used to perform co-culture experiments.

### 2.2 Peripheral blood mononuclear cell isolation

Human PBMC were isolated from buffy coats (Dutch blood bank, Amsterdam, The Netherlands). Buffy coats from healthy donors were diluted (1:1) in PBS supplemented with 2% FCS. PBMC fraction was isolated by density gradient centrifugation (1000 x g, 13 minutes) using Leucosep tubes (Greiner Bio-one). After washing, remaining red blood cells were lysed (4.14 g NH_4_Cl, 0.5 g KHCO_3_, 18.6 mg Na_2_EDTA in 500 mL demi water, sterile filtered, pH = 7.4). Purified PBMC were resuspended in RPMI 1640 supplemented with 2.5% FCS, penicillin (100 U/mL) and streptomycin (100 µg/mL) and used for IEC/PBMC co-culture experiments.

### 2.3 IEC/PBMC co-culture

FHs 74 Int and HT-29 were grown in transwell inserts (12 well inserts with 0.4 µm pore polyester membrane) and exposed apically to a 1:1 mixture of 2’FL and GF in 0.25-1% solution (2.5-10 mg/mL, *w/v*%) in the presence of CpG (M362, 0.1 or 0.5 µM, Invivogen). In the basolateral compartment 2 x 10^6^ cell/mL PBMC were added (1.5 mL), activated with αCD3/αCD28 (0.15 µg/mL and 0.2 µg/mL respectively from Sanquin and BD Biosciences, San Jose, CA, USA), and incubated at 37 °C and 5% CO_2_ for 24 hours. Alternatively, before exposure to 2’FL/GF and CpG, IEC were pre-incubated with 10 µM GW4869 (CAS Number 6823-69-4, Sigma-Aldrich, St. Louis, MO, USA) for 1 hour before basolateral exposure to activated PBMC. After 24 h of incubation, the basolateral supernatant was collected and stored at -20 °C for cytokine and galectin analysis.

Then, IEC were separated from the PBMC by transferring the inserts to a new plate, washed with PBS and incubated in fresh medium in the presence or absence OF 10 µM GW4869 for additional 24 hours to study the basolateral IEC-derived galectin release. Additionally, the basolateral epithelial cell culture supernatant (conditioned supernatant) was used to isolate HT-29 derived exosomes. An illustration describing the IEC/PBMC co-culture model is available in **Supplementary figure 1**.

### 2.4 Exosome isolation

Exosomes were isolated from conditioned supernatant (13.5 mL) using ExoQuick-TC™ ULTRA Isolation Kit (System Biosciences LLC, Palo Alto, CA, USA). Exosomes were also isolated from plain RPMI (supplemented with 2.5% FCS and penicillin/streptomycin) as a control. Shortly, conditioned supernatant was collected and after cell debris removal by spinning down (3000 x g, 15 minutes) ExoQuick-TC was added and incubated overnight at 4 °C. Upon centrifugation (3000 x g, 10 minutes, 4 °C) the exosome pellet was collected and purified following manufacturer’s instructions. After purification, the protein concentration was measured by Nanodrop One (Thermo Fisher scientific) after which purified exosomes were stored at −80 °C for further analysis.

### 2.5 Enzyme-linked immunosorbent assay

The cytokine and galectin secretion was studied in the basolateral supernatant of IEC/PBMC co-cultures. Commercially available kits were used to measure IFNγ, IL-13, TNFα (all from Thermo Fisher Scientific, Waltham, MA, USA), IL-10 (U-Cytech, Utrecht, The Netherlands), and galectin-3 (R&D systems) following manufacturer’s protocol. Human galectin-4 and -9 were measured using antibody pairs (all from R&D systems, Minneapolis, MN, USA) as described before (9, 26, 27). Additionally, a CD63 detection ELISA kit (System Biosciences) was used to measure the exosomes isolated from conditioned supernatant following manufacturer’s protocol.

### 2.6 Western blot

Isolated exosome samples (20 µl/sample; 8-10 µg exosomes, aroud 40 µg lysate and 20 pg recombinant galectins) were mixed with Laemmli sample buffer (Bio-rad) containing 50 mM dithiothreitol (DTT) and incubated at 95 °C for 5 minutes to reduce and denaturate proteins before loading into a 4-20% SDS-PAGE gel (Mini-PROTEAN^®^ Bio-Rad, Veenendaal, The Netherlands) for separation by electrophoresis. Separated proteins were then transferred into a polyvinylidene difluoride membrane (Transblot Turbo, Bio-Rad) after which the membrane was blocked with 5% milk protein in phosphate-buffered saline containing 0.05% Tween-20. The membranes were then incubated with galectin-4 (1:4000) and galectin-9 (1:100) antibodies (both from R&D). After overnight incubation, the membranes were washed and incubated with horseradish peroxidase-conjugated secondary antibodies (1:5000, Dako, Santa Clara, CA, USA) for 2 h. After washing, ECL reagent (Cytiva, Marlborough, MA, USA) was used to visualize the proteins. Image J software (Wayne Rasband, National Institutes of Health, Bethesda, MD, USA) was used to analyze the data.

### 2.7 Statistical analysis

All statistical analysis were done using GraphPad Prism software (San Diego, CA, USA). Data were analyzed using one-way ANOVA followed by Bonferroni’s multiple comparison *post hoc* test. When data did not fit normal distribution, transformation was applied prior to ANOVA analysis. In FHs 74 Int co-cultures the conditions with different concentrations of CpG were analyzed separately as represented by the dotted line. However, within the analysis of CpG-exposed conditions a comparison between medium control and CpG alone was included. Probability values of *p* < 0.05 were considered significant.

## 3 Results

### 3.1 FHs 74 Int/PBMC co-culture results

To study if the FHs 74 Int IEC had the ability to crosstalk with immune cells in an IEC/PBMC co-culture model, FHs 74 Int were seeded in transwell inserts and co-cultured with non-stimulated or αCD3/CD28-activated PBMC for 24 hours alone or in combination with CpG (0.5 µM). Additionally, after FHs 74 Int co-culture with PBMC, FHs 74 Int were washed and incubated in fresh medium for additional 24 h after which epithelial-derived galectin-9 was measured.

**Figure 1:**
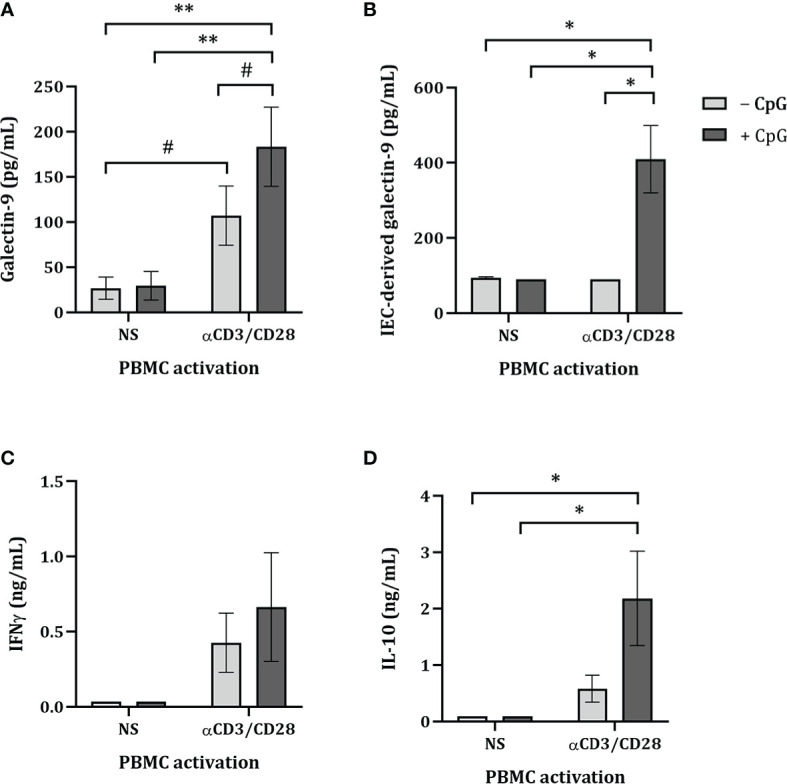
Cytokine and galectin-9 secretion in FHs 74 Int/PBMC co-culture model after exposure to non-activated or activated PBMC. FHs 74 Int IEC were stimulated with CpG (0.5 µM) and basolaterally exposed to non-activated (ns) or αCD3/CD28-activated-PBMC for 24 h. After incubation, galectin-9 **(A)**, IFNγ **(C)** and IL-10 **(D)** were measured in the basolateral supernatant. Additionally, after IEC/PBMC co-culture, FHs 74 Int were washed and incubated in fresh medium for additional 24 h (total 48h; 24h IEC/PBMC co-culture and additional 24h IEC culture) after which IEC-derived galectin-9 **(B)** was measured in the basolateral supernatant. Data represent mean ± SEM of *n* = 3 independent PBMC donors (# *p* < 0.1, *p < 0.05, **p < 0.01).

Upon exposure to CpG there was no effect on the levels of galectin-9, IFNγ, IL-10 and IEC-derived galectin-9 secretion of non-stimulated PBMC (**Figures 1A–D**). Co-culture of FHs 74 Int with αCD3/CD28-activated PBMC tended to increase galectin-9 secretion (*p* = 0.06) but did not affect the levels of IFNγ, IL-10 and IEC-derived galectin-9 secretion as compared to non-stimulated PBMC (**Figures 1A–D**). However, exposure of FHs 74 Int to CpG and αCD3/CD28-activated PBMC significantly increased galectin-9, IL-10 and IEC-derived galectin-9 secretion as compared to non-stimulated or αCD3/CD28-activated PBMC (**Figures 1A–D**).

Since only upon stimulation of PBMC with αCD3/CD28 upregulated cytokine secretion is observed, which can be modulated by stimulation with CpG, the following studies were done using only αCD3/CD28-activated PBMC in co-culture with IEC.

### 3.2 NDO modulate cytokine secretion from FHs 74 Int

By means of the FHs 74 Int and PBMC co-culture model, the ability of NDO in combination with CpG to induce immunomodulatory effects was studied. Therefore, FHs 74 Int were exposed to 2’FL, GF and a 1:1 mixture of 2’FL and GF (0.5% *w/v*) in combination with 0.1 or 0.5 µM CpG for 24 h. Additionally, after FHs 74 Int co-culture with PBMC, FHs 74 Int were washed and incubated in fresh medium for additional 24 h after which epithelial-derived galectin-9 was measured.

**Figure 2:**
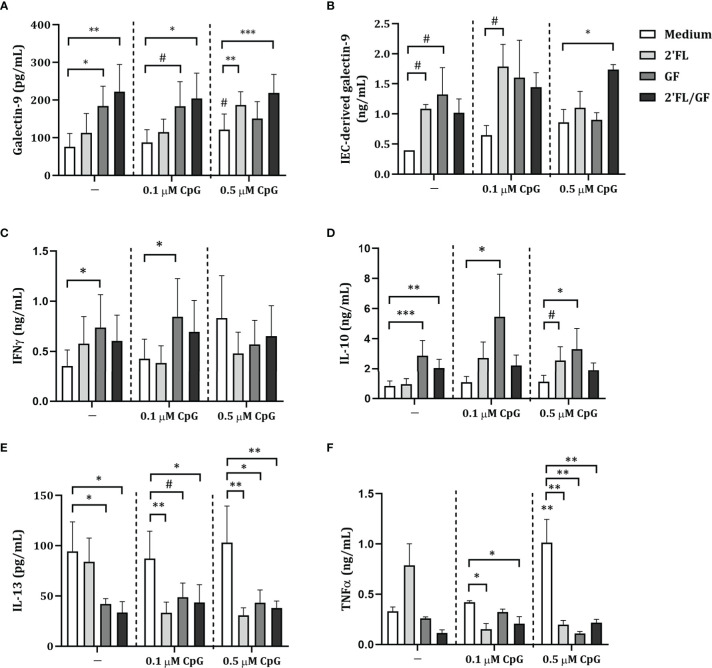
Cytokine and galectin-9 secretion in FHs 74 Int/PBMC co-culture model after exposure to non-digestible oligosaccharides and CpG. FHs 74 Int IEC were stimulated with 2’FL, GF or 1:1 mixture of 2’FL and GF (0.5% *w/v*) in combination with CpG (0.1 or 0.5 µM) and basolaterally exposed to αCD3/CD28-activated PBMC for 24 h. After incubation, galectin-9 **(A)**, IFNγ **(C)**, IL-10 **(D)**, IL-13 **(E)** and TNFα **(F)** were measured in the basolateral supernatant. Additionally, after IEC/PBMC co-culture, FHs 74 Int were washed and incubated in fresh medium for additional 24 h (total 48h; 24h IEC/PBMC co-culture and additional 24h IEC culture) after which IEC-derived galectin-9 **(B)** was measured in the basolateral supernatant. Data represent mean ± SEM of *n* = 3 independent PBMC donors. The conditions without CpG and with 0.1 or 0.5 µM CpG were analyzed separately as represented by the dotted line. Within this analysis, a comparison between the medium control and CpG alone was included (# *p* < 0.1, * *p* < 0.05, **p < 0.01, ***p < 0.001).

There was no effect on galectin-9, IFNγ, IL-10, IL-13 or TNFα (**Figures 2A, C–F**) secretion of FHs 74 Int exposed to 2’FL but IEC-derived galectin-9 secretion tended to increase (*p* = 0.07) (**Figure 2B**). Exposure to GF alone significantly increased galectin-9, IFNγ, IL-10 and tended to increase IEC-derived galectin-9 (*p* = 0.07), while significantly decreasing IL-13 levels (**Figures 2A–E**). Significantly increased galectin-9 and IL-10, and decreased IL-13 concentrations were observed upon exposure of FHs 74 Int to a 1:1 mixture of 2’FL and GF (**Figures 2A, D, E**).

Exposure to 0.1 or 0.5 µM CpG did not affect galectin-9, IEC-derived galectin-9, IFNγ, IL-10, IL-13 or TNFα, except for significantly increased TNFα and a tendency towards increased galectin-9 concentrations (*p* = 0.08) upon exposure to 0.5 µM CpG as compared to medium control (**Figures 2A–F**). Combined exposure to both concentrations of CpG and 2’FL significantly decreased IL-13 and TNFα concentrations (**Figures 2E, F**). Combined exposure to 2’FL and 0.5 µM CpG significantly increased galectin-9 and tended to increase IL-10 (*p* = 0.06) concentrations (**Figure 2A, D**). IEC-derived galectin-9 tended to increase only upon exposure to 2’FL and 0.1 µM CpG (*p* = 0.09) (**Figure 2B**). IEC-derived galectin-3 tended to increase upon exposure to 2’FL and 0.1 µM CpG and significantly increased with GF and 0.1 µM CpG as compared to 0.1 µM CpG alone (**Supplementary figure 2**). IEC-derived galectin-4 was under detection limit.

Exposure to GF and 0.1 µM CpG significantly increased IFNγ and IL-10 and tended to increase galectin-9 (*p* = 0.06) and decrease IL-13 (*p* = 0.09) concentrations (**Figures 2A, D, E**). Meanwhile, when GF was combined with 0.5 µM CpG significantly increased IL-10 and decreased IL-13 and TNFα concentrations were observed (**Figures 2D–F**).

There was no effect on IFNγ and IL-10 concentrations upon exposure to 2’FL/GF and CpG (in both concentrations) (**Figures 2C, D**). However, significantly increased galectin-9 and decreased IL-13 and TNFα concentrations were observed upon exposure to 2’FL/GF and CpG (in both concentrations) (**Figures 2A, E, F**). Only when 2’FL/GF was combined with 0.5 µM CpG significantly increased IEC-derived galectin-9 concentrations were observed (**Figure 2B**).

Taken together, exposure of FHs 74 Int and PBMC to NDO alone significantly modulated cytokine and galectin-9 release. Combined exposure to NDO and CpG further modulated the cytokine secretion from FHs 74 Int and PBMC co-cultures. Since exposure to 2’FL/GF in combination with CpG significantly increased galectin-9 and epithelial-derived galectin-9 as well as decreasing Th2-type IL-13 and pro-inflammatory TNFα, following studies were done focusing on the 1:1 mixture of 2’FL/GF.

### 3.3 Increased IEC-derived galectin-9 secretion upon exposure to 2’FL/GF and CpG

To further study the involvement of IEC-derived galectins in the modulation of cytokine secretion in the IEC/PBMC co-culture model, following studies were done using HT-29 IEC since these were shown to secrete not only IEC-derived galectin-9 but also galectin-3 and -4 upon stimulation with NDO and CpG (9). Therefore, IEC (HT-29 cell line) were apically exposed to 2’FL/GF mixture in combination with 0.5 µM CpG and basolaterally to αCD3/CD28-activated PBMC for 24 h. After IEC/PBMC co-culture, IEC were separated from PBMC, washed and incubated in fresh medium for additional 24 h to study the IEC-derived galectin secretion.

First a dose-response experiment was done using 0.25-1% (*w/v*) 2’FL/GF to determine the optimal concentration at which the most relevant immunomodulatory effects are observed. Results showed that in association with CpG exposure to 0.5% 2’FL/GF was enough to promote regulatory IL-10 and IEC-derived galectin-9 as well as decreased concentrations of IL-13 and a tendency towards increased IFNγ (**Supplementary figure 3**). Thus, following studies were done using 0.5% 2’FL/GF.

**Figure 3:**
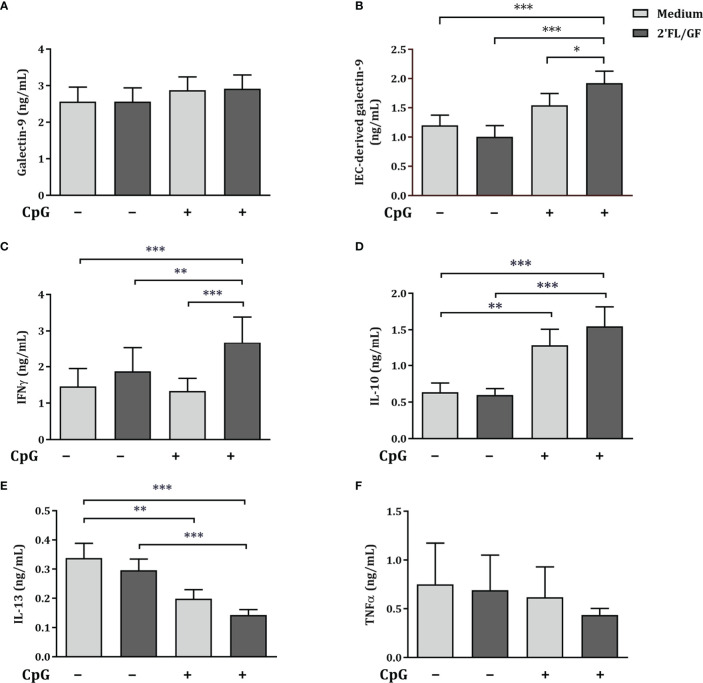
Cytokine and galectin-9 secretion in IEC/PBMC co-culture. IEC (HT-29 cell line) were stimulated with a 1:1 mixture of 2’FL and GF (0.5% *w/v*) in combination with CpG (0.5 µM) and basolaterally exposed to αCD3/CD28-activated PBMC for 24 h. After incubation, galectin-9 **(A)**, IFNγ **(C)**, IL-10 **(D)**, IL-13 **(E)** and TNFα **(F)** were measured in the basolateral supernatant. Additionally, after IEC/PBMC co-culture, HT-29 IEC were washed and incubated in fresh medium for additional 24 h (total 48h; 24h IEC/PBMC co-culture and additional 24h IEC culture) after which IEC-derived galectin-9 **(B)** was measured in the basolateral supernatant. Data are represented as mean ± SEM of *n* = 6 **(E)**, *n* = 7 **(A, B, E)** or *n* = 8 **(A, C, D)** independent PBMC donors (# p < 0.1, *p < 0.05, **p < 0.01, ***p < 0.001).

Exposure to 2’FL/GF alone did not affect galectin-9, epithelial-derived galectin-9, IFNγ, IL-10, IL-13 and TNFα concentrations (**Figures 3A–F**). Exposure to CpG alone significantly increased IL-10 and decreased IL-13 concentrations as compared to medium control (**Figures 3D, E**). Combined exposure to 2’FL/GF and CpG, significantly increased IEC-derived galectin-9, IFNγ and IL-10, and decreased IL-13 concentrations, without affecting TNFα, as compared to medium control, 2’FL/GF alone and/or CpG alone (**Figures 3B–F**).

Altogether, Th1-type IFNγ and regulatory type IL-10 and IEC-derived galectin-9 were significantly increased, and Th2-type IL-13 was decreased suggesting Th1-type regulatory immunomodulatory activity of 2’FL/GF and CpG in the IEC/PBMC model.

Besides IEC-derived galectin-9 secretion, the secretion of galectin-3 and -4, were studied upon exposure of HT-29 and PBMC to 2’FL/GF and CpG. There was no effect on IEC-derived galectin-3 concentrations (**Supplementary figure 4**). Exposure to 2’FL/GF or CpG alone did not affect the concentrations of IEC-derived galectin-4. However, upon combined exposure to 2’FL/GF and CpG, significantly increased IEC-derived galectin-4 secretion was observed (**Supplementary figure 4**).

In order to study the connection between epithelial-derived galectin secretion and the cytokine secretion in the IEC/PBMC co-culture correlation analysis were done. The correlation coefficient (r) and the probability values (*p*) are summarized in **Table 1** and the correlation plots are shown in **Supplementary figure 5**. Th1-type IFNγ secretion was positively correlated with epithelial derived galectin-3, and -4. Epithelial-derived galectin-4 and -9, but not galectin-3 correlated positively with IL-10 secretion. Meanwhile, only epithelial-derived galectin-9 correlated negatively with IL-13 secretion. Although in this experiment, no correlation was observed between IFNγ secretion and IEC-derived galectin-9, in the correlations of the dose-response experiments, a positive correlation was observed (**Supplementary figure 3**).

**Table 1:** Correlations between cytokines in the IEC/PBMC co-culture and epithelial-derived galectins.

	IFNγ	IL-10	IL-13
**IEC-derived galectin-3**	+ r = 0.06 *p* = 0.001 (**)	n.s. r = 0.17 *p* = 0.4	n.s. r = − 0.33 *p* = 0.11
**IEC-derived galectin-4**	+ r = 0.45 *p* = 0.01 (*)	+ r = 0.43 *p* = 0.015 (*)	n.s. r = 0.41 *p* = 0.045
**IEC-derived galectin-9**	n.s. r = 0.21 *p* = 0.3	+ r = 0.67 *p* < 0.001 (***)	− r = − 0.7 *p* = 0.0002 (***)

(−) negative correlation; (n.s.) non-significant correlation; (+) positive correlation.

The correlation plots are shown in **Supplementary figure 5**. * p < 0.05.** p < 0.01.*** p < 0.001.

Epithelial-derived galecin-9 concentrations were significantly increased upon exposure to 0.5% 2’FL/GF and CpG after IEC/PBMC co-culture. Exposure of HT-29 to CpG and 0.5% 2’FL/GF significantly increased Th1-type IFNγ, and regulatory IL-10 and epithelial-derived galectin-9, while decreasing Th2-type IL-13. The cytokine secretion in IEC/PBMC co-culture significantly correlated with epithelial-derived galectin-9 secretion, which suggests that epithelial-derived galectin-9 is involved in the immunomodulatory effects observed upon exposure to 2’FL/GF and CpG in the IEC/PBMC co-culture model.

### 3.4 Galectins are released by HT-29 through exosomes in IEC/PBMC co-culture

**Figure 4:**
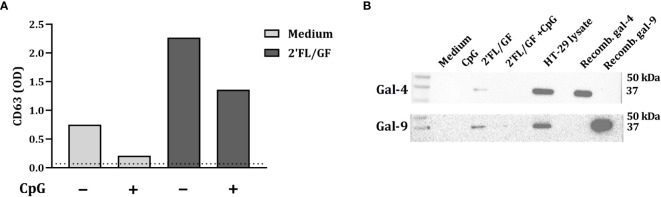
Galectin-4 and -9 are present in CD63-expressing exosomes. IEC (HT-29 cell line) were apically exposed to 0.5% (*w/v*) 2’FL/GF alone or in combination with 0.5 µM CpG and basolaterally to αCD3/CD28-activated PBMC. After 24 h incubation, IEC were separated from PBMC, washed and incubated in fresh medium for additional 24 h (total 48h; 24h IEC/PBMC co-culture and additional 24h IEC culture) after which the basolateral supernatant was collected and exosomes isolation was performed. The presence of exosomes was confirmed by measuring CD63 marker **(A)** by means of ELISA. The dotted line represent CD63 present in culture medium (non-exposed to IEC). Additionally, the presence of galectin-4 and -9 within CD63-expressing exosomes was studied by western blot **(B)** of *n* = 1.

Exosomes are implicated in cell-to-cell communication and galectins have relevant roles in modulating several immune processes. In order to study if HT-29 secrete galectins *via* exosomes, after exposure to NDO and CpG, IEC-derived supernatant was collected and exosomes were isolated. To confirm the presence of exosomes in the supernatant, CD63 expression was measured by ELISA. As a control, exosomes were isolated from RPMI medium supplemented with FCS, which gave the lowest CD63 signal (represented as dotted line) (**Figure 4A**). In IEC supernatants from IEC/PBMC co-cultures conditioned with medium and CpG, increased CD63 secretion was observed. This was further increased by the addition of 2’FL/GF (**Figure 4A**).

Additionally, the presence of galectin-4 and -9 in the exosomes was studied by western blot. Galectin-4 and -9 expression was found in 2’FL/GF stimulated exosome samples (**Figure 4B**). However, galectin-4 and -9 were not detected in medium samples or in samples exposed to CpG alone or in combination with 2’FL/GF (**Figure 4B**).

### 3.5 Exposure to GW4869 suppressed IEC-derived galectin-4 and -9 secretion

To study how IEC secrete galectins, we apically exposed IEC (HT-29 cell line) to 10 µM GW4869, the neutral sphingomyelinase inhibitor which functions as an inhibitor of the exosome biogenesis. After 1h incubation we exposed IEC to CpG alone or in combination with 0.5% 2’FL/GF and co-cultured with activated-PBMC. After 24 h co-culture, IEC were separated, washed and incubated again with 10 µM GW4869 for 24 h after which IEC supernatant was collected for epithelial-derived galectin measurement.

**Figure 5:**
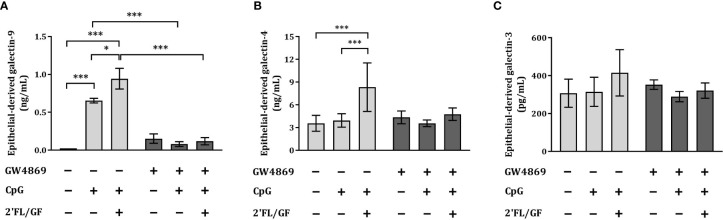
Exposure to GW4869 suppressed epithelial-derived galectin-4 and -9 secretion. IEC (HT-29 cell line) were apically exposed to 10 µM GW4869 for 1 hour after which 0.5% (*w/v*) 2’FL/GF mixture and CpG were added. In the basolateral compartment αCD3/CD28-activated PBMC were added and incubated. After 24h incubation, IEC were separated from the PBMC fraction, washed and incubated in fresh medium or in the presence of 10 µM GW4869 for additional 24 h (total 48h; 24h IEC/PBMC co-culture and additional 24h IEC culture) after which the basolateral supernatant was collected and epithelial-derived galectin-9 **(A)**, -4 **(B)** and -3 **(C)** were measured. Data are represented as mean ± SEM of *n* = 6 **(A, B)** or *n* = 4 **(C)** independent PBMC donors (*p < 0.05, ***p < 0.001).

IEC-derived galectin-9 was significantly increased upon exposure to CpG alone or in combination with 0.5% 2’FL/GF as compared to medium control and/or CpG alone (**Figure 5A**). Exposure to GW4869 suppressed IEC-derived galectin–9 secretion as observed upon exposure to CpG alone or 2’FL/GF and CpG (**Figure 5A**). There was no effect on IEC-derived galectin-3 and -4 upon exposure to CpG alone or in combination with 2’FL/GF, and IEC-derived galectin-4 was significantly increased only upon exposure to 0.5% 2’FL/GF and CpG, as compared to medium control or CpG alone (**Figures 5B, C**). In the presence of GW4869, the increase in epithelial-derived galectin-4 secretion induced by 2’FL/GF and CpG was suppressed. This might indicate that GW4869 may have partially contributed to blocking 2’FL/GF and CpG induced intestinal-epithelial galectin-4 secretion, although this effect did not reach significance when comparing both groups.

The secretion of IEC-derived galectin-9 induced by 2’FL/GF and CpG was suppressed upon exposure to GW4869 inhibitor.

### 3.6 Exposure to GW4869 prevented the rise in IL-10 and downregulation in IL-13 secretion in IEC/PBMC co-culture

To study the effects of the inhibitor of exosome biogenesis GW4869 in the cytokine secretion in HT-29/PBMC co-culture, we studied the secretion of IFNγ, IL-13 and IL-10 concentrations upon exposure to CpG and 2’FL/GF in the presence or absence of 10 µM GW4869.

**Figure 6:**
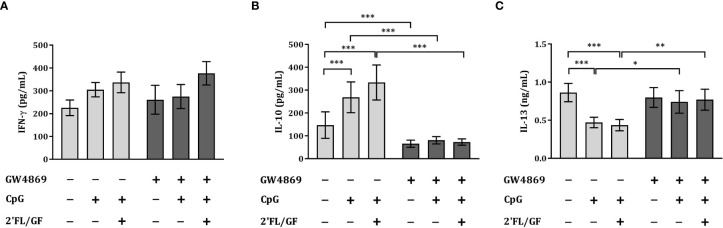
Exposure to GW4869 prevented the increase in IL-10 and the reduction in IL-13 secretion. IEC (HT-29 cell line) were apically exposed to 10 µM GW4869 for 1 hour after which 0.5% (*w/v*) 2’FL/GF mixture and 0.5 µM CpG were added. In the basolateral compartment αCD3/CD28-activated PBMC were added and incubated. After 24 h incubation, the basolateral supernatant was collected and IFNγ **(A)**, IL-10 **(B)** and IL-13 **(C)** were measured. Data are represented as mean ± SEM of *n* = 6 **(B,C)** or *n* = 5 **(A)** independent PBMC donors (*p < 0.05, **p < 0.01, ***p < 0.001).

There was no effect on IFNγ concentrations upon exposure to CpG alone or in combination with 2’FL/GF (**Figure 6A**). However, significantly increased IL-10 and decreased IL-13 concentrations were found upon exposure to CpG alone or in combination with 2’FL/GF as compared to medium control (**Figures 6B, C**). When IEC were exposed to GW4869 in the presence of 2’FL/GF and CpG, and co-cultured with activated-PBMC, the reduction in IL-13 secretion as well as the increase in IL-10 concentrations was suppressed (**Figures 6B, C**).

The reduction in Th2-type IL-13 and increase in regulatory type IL-10 promoted upon exposure to 2’FL/GF and CpG was suppressed upon exposure of IEC to the exosome biogenesis inhibitor GW4869.

## 4 Discussion

Galectins are soluble type lectins secreted by IEC and immune cells which have relevant functions in regulating immune responses (10, 28, 29). In particular, IEC-derived galectin-9 was identified as a key factor contributing to immune regulation in an *in vitro* co-culture model combining IEC and immune cells (2, 3, 9, 30) as well as *in vivo* in a murine food allergy prevention model (5), which was secreted upon exposure to NDO and a bacterial trigger such as TLR9 agonist. In these *in vitro* studies, the HT-29 cell line was used as a model of intestinal epithelial cells.

### 4.1 Primary FHs 74 Int or HT-29 in IEC/PBMC co-culture

Our aim was to study whether a non-carcinogenic intestinal epithelial cell line would be able to modulate immune effects of NDO and CpG, similar to what was previously shown for HT-29 cell line (2, 3, 9). Therefore, the human fetal intestinal cell line FHs 74 Int was used in co-culture with human PBMC. In line with previous studies, only upon activation of PBMC, CpG-induced upregulation of Th1-type IFNγ, regulatory IL-10 and galectin-9 were found in IEC/PBMC co-culture model (9, 31). The addition of NDO enhanced these Th1-type and regulatory responses while lowering Th2-type IL-13 secretion (2, 3, 9, 27). Unlike in the HT-29 cell line, in the FHs 74 Int/PBMC co-culture, already in the absence of CpG, GF or 2’FL/GF alone were able to modulate the secretion of cytokines like Th1-type IFNγ, Th2-type IL-13 as well as regulatory IL-10 and galectin-9. This suggests that primary epithelial cells such as the FHs 74 Int are more responsive to NDO exposure than the carcinogenic HT-29 cell line. The presence of an inflammatory environment represented by the activated-PBMC in co-culture with the FHs 74 Int which have an immature phenotype, as opposed to HT-29 which are derived from adult, might have promoted the secretion of cytokines already upon exposure to NDO alone. Other studies also indicated that FHs 74 Int cell line was more susceptible to inflammation than other adult cell lines (32, 33). These results should be further confirmed with additional experiments using this or other more complex *in vitro* models.

In addition, when NDO were combined with CpG and exposed to the FHs 74 Int/PBMC co-culture, upregulated regulatory-type Th1 and downregulated Th2-type cytokines were observed, similar to what was previously observed in HT-29 or T84 and PBMC co-cultures (2, 9). However, even though the HT-29 cells were found to release also galectin-4 and galectin-3 beyond galectin-9 (2, 9, 27), the primary FHs 74 Int epithelial cells only secreted galectin-9 and -3, but not galectin-4. Also others found anti-inflammatory effects in primary fetal epithelial cells using NDO. Downregulated IL-8 secretion was observed in TNFα-activated FHs 74 Int exposed to NDO (34). Furthermore, in FHs 74 Int exposed to human milk, growth-related effects of immature epithelial cells (32) and regulation of inflammatory responses (33) were observed. This supports the ability of bioactive components in human milk such as human milk oligosaccharides (HMOS) to play a role in IEC maturation and regulation of IEC function. These studies support the potential use of FHs 74 Int primary IEC *in vitro* to study the modulation of immune responses upon exposure to NDO. Other non-carcinogenic human intestinal epithelial cell lines are also available such as HIEC-6, H4 or NCM-460. However, the FHs 74 Int is a primary human intestinal cell line isolated from the fetal small intestine, it has been validated as a model of intestinal epithelium in newborns (33) which is of interest to study the regulation of immune responses in early life.

### 4.2 Galectins and immune-regulation

The immunomodulatory properties of a mixture of 2’FL and GF in combination with CpG observed in the present study are in line with previous studies (2, 3, 9). Exposure of HT-29 to 2’FL/GF resulted in upregulated Th1-type IFNγ and regulatory epithelial-derived galectin-9. The increased secretion of epithelial-derived galectins was described before upon exposure to 2’FL or GF in association with CpG (9). Also in the current study, IEC-derived galectin-9, -4 and -3 secretion was positively correlated with IFNγ, while IEC-derived galectin-9 and -4 were positively correlated with IL-10, and IEC-derived galectin-9 negatively correlated with IL-13 secretion in the HT-29/PBMC co-culture. These correlations were particularly pronounced for IL-10 and IL-13 and IEC-derived galectin-9, which emphasizes the involvement of epithelial-derived galectins in supporting immunomodulation. These results are in line with previous studies where exposure of αCD3/CD28-activated PBMC to recombinant galectin-9 significantly increased IFNγ and IL-10 secretion in the IEC/PBMC co-culture (5). In addition, neutralization of galectin-9 using Tim3Fc fusion protein prevented the increase in IFNγ and IL-10 secretion in IEC/PBMC (2). These studies demonstrate the involvement of epithelial-derived galectin-9 in promoting immunomodulation.

Similar to the results with the HT-29 cells, also FHs 74 Int primary IEC show the same immunomodulatory effects already in the absence of CpG exposure, modulating galectin-9 secretion, therefore we hypothesize that galectin-9 has a major role in mucosal immune modulation by NDO. In a murine food allergy model, NDO were found to enhance galectin-9 expression in IEC, while increasing the serum levels in association with modulation of the mucosal immune response and allergy protection (5). Others have also previously described immunomodulatory properties of galectins (28, 35–39) with roles in epithelial homeostasis (40, 41) which emphasizes the contribution of epithelial-mediators such as galectins in regulation of mucosal immune responses.

### 4.3 Galectin secretion *via* exosomes

Little is known about the cellular mechanisms involved in the secretion of galectins and how these can be influenced by NDO. Since galectins lack a N-terminal signaling sequence to direct them through the endoplasmic reticulum for secretion, un-conventional transport pathways are thought to be the routes used for galectin secretion (11). One of the routes by which galectins are secreted into the extracellular milieu are extracellular vesicles (11, 21). Although it’s still unknown how galectins are recruited into extracellular vesicles, various studies have demonstrated the localization of galectin-3, -4 and -9 inside multivesicular bodies (11, 21, 22).

Here we show that CD63-expressing exosomes were released by the IEC into the basolateral compartment after IEC/PBMC co-culture, and these exosomes were found to contain galectin-9 and -4 as measured by western blot. Depending on the experimental condition, the amount of exosomes isolated differed which might have hampered the detection of galectin-9 and -4 for all conditions. As was shown previously using the HT-29/PBMC model (27), NDO facilitate CpG induced galectin-9 release by IEC and this may have taken place during the first 24 h, thus during the co-culture with PBMC. The CpG and 2’FL/GF condition may therefore have already caused the exosome release which might explain a more limited signal observed after the additional 24 h of epithelial cell culture following the co-culture with PBMC. However, since galectins were measured in the IEC supernatant by means of ELISA, also in 2’FL/GF and CpG condition, we hypothesize that in all conditions the IEC-derived galectins were secreted *via* exosomes, similar to what was observed for the 2’FL/GF condition. Further studies should be performed in order to confirm that upon exposure to CpG alone or in combination with 2’FL/GF epithelial-derived galectins are also present in exosomes which should include the quantification of the levels of galectins per exosome.

To study if exosomes were involved in the mechanism by which NDO and CpG promoted the secretion of galectins, IEC were exposed to GW4869, a specific neutral sphingomyelinase (nSMase) inhibitor known to block the exosome biogenesis and thereby their release (26). GW4869 in particular suppressed epithelial-derived galectin-9, indicating that the inhibition of exosome biogenesis by GW4869 might have hampered the recruitment of galectin-9, and in part galectin-4, into the multivesicular bodies as well as their release into the basolateral compartment. However, galectin-3 secretion was not affected by 2’FL/GF and CpG which suggests that only the secretion of the newly formed galectins was inhibited by GW4869, but not the constitutive secretion of galectin-3 from IEC. It remains to be confirmed whether the yield of exosome secretion was lower in GW4869-exposed conditions as compared to non-exposed conditions. Although most of the galectin-9 secretion was blocked by exposure to exosome biogenesis inhibitor GW4869, it can’t be excluded that the residual galectin-9 secretion is derived from other secretion mechanisms such as through direct transport across the membrane (11).

### 4.4 Involvement of galectins on immunomodulation

The secretion of epithelial-derived galectin-9 were positively correlated with IL-10 and negatively with IL-13 secretion in the IEC/PBMC co-culture. Blocking the galectin-9 release induced by 2’FL/GF and CpG through the inhibition of exosomes secretion from IEC was found to prevent the increase of IL-10 secretion, while blocking the reduction in IL-13 secretion. These results, imply an essential role for epithelial-derived galectin-9 in the regulation of the immunomodulatory effects observed in the IEC/PBMC co-culture upon exposure to NDO and CpG. These results are in accordance with a previous study showing the essential immunomodulatory role for galectin-9 in this co-culture model in which the use of either lactose or Tim3Fc fusion protein to block galectin-9 resulted in suppression of CpG and NDO induced IFNγ and IL-10 secretion (2).

In conclusion, exposure to NDO promoted Th1-type regulatory effects in IEC, while driving away from the Th2 phenotype, in association with CpG in the HT-29/PBMC co-culture. In the primary FHs 74 Int/PBMC co-culture the NDO induced similar immunomodulatory effects already in the absence of CpG. Galectin-9 and -4 were present in epithelial-derived exosomes and inhibition of exosome biogenesis by IEC inhibited galectin release, blocking the immunomodulatory effects of 2’FL/GF and CpG. This demonstrates an essential role for galectin-9 from IEC-derived exosomes in NDO induced mucosal immune regulation.

## Data availability statement

The original contributions presented in the study are included in the article/**Supplementary Material**. Further inquiries can be directed to the corresponding author.

## Author contributions

Conceptualization and methodology, VA-M, AK, BvL and LW; investigation, VA-M, MB, BB, AB; writing-original draft preparation, VA-M, BvL and LW; writing-review and editing, VA-M, MB, BB. AB., JG, AK, BvL and LW; visualization, VA-M; supervision and project administration, BvL and LW. All authors contributed to the article and approved the submitted version.
