# Supplementary material for: Epithelial-derived galectin-9 containing exosomes contribute to the immunomodulatory effects promoted by 2’-fucosyllactose and short-chain galacto- and long-chain fructo-oligosaccharides
Source: Front Immunol. 2022 Dec 22;13:1026031. doi: 10.3389/fimmu.2022.1026031 (PMC9846635; doi:10.3389/fimmu.2022.1026031)

Supplementary figures

Supplementary figure 1. IEC/PBMC co-culture model description. IEC (FHs 74 Int or HT-29) were grown in transwell inserts until confluency and exposed to 2’FL, GF or a mixture of both (0.5% *w/v*) in the presence or absence of CpG (0.1 or 0.5 µM), and basolaterally co-cultured with non-activated or αCD3/CD28-activated PBMC (**B**). Alternatively, to block exosome secretion 1 h before NDO and CpG exposure, IEC were pre-incubated with GW4869 (10 µM) (**A**). After IEC/PBMC co-culture, IEC were transferred into a new plate, washed and incubated for additional 24 h (48 h in total; 24h in IEC/PBMC co-culture and additional 24 h IEC culture) in fresh medium alone or in the presence of GW4869 (**C**).

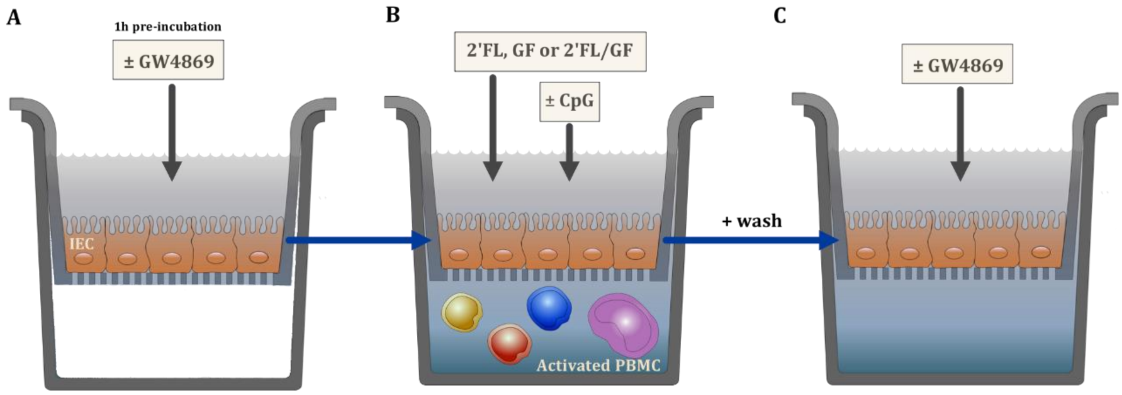

Supplementary figure 2. IL-13, TNFα and galectin-3 secretion in FHs 74 Int/PBMC co-culture model. FHs 74 Int IEC were stimulated with CpG (0.1 or 0.5 μM) in the presence of 2'FL, GF or a 1:1 mixture of 2'FL and GF (0.5% *w/v*). Basolaterally non-activated (NS) or αCD3/CD28-activated PBMC were added and incubated for 24 h after which IL-13 (**A**) and TNFα (**B**) concentrations were measured. Then, FHs 74 Int were washed and incubated in fresh medium for additional 24 h (48 h in total; 24 h IEC/PBMC co-culture and additional 24 h of IEC culture) after which IEC-derived galectin-3 was measured (**C-D**). Data represent mean ± SEM of n = 3 independent PBMC donors (# $p < 0.1$, * $p < 0.05$).

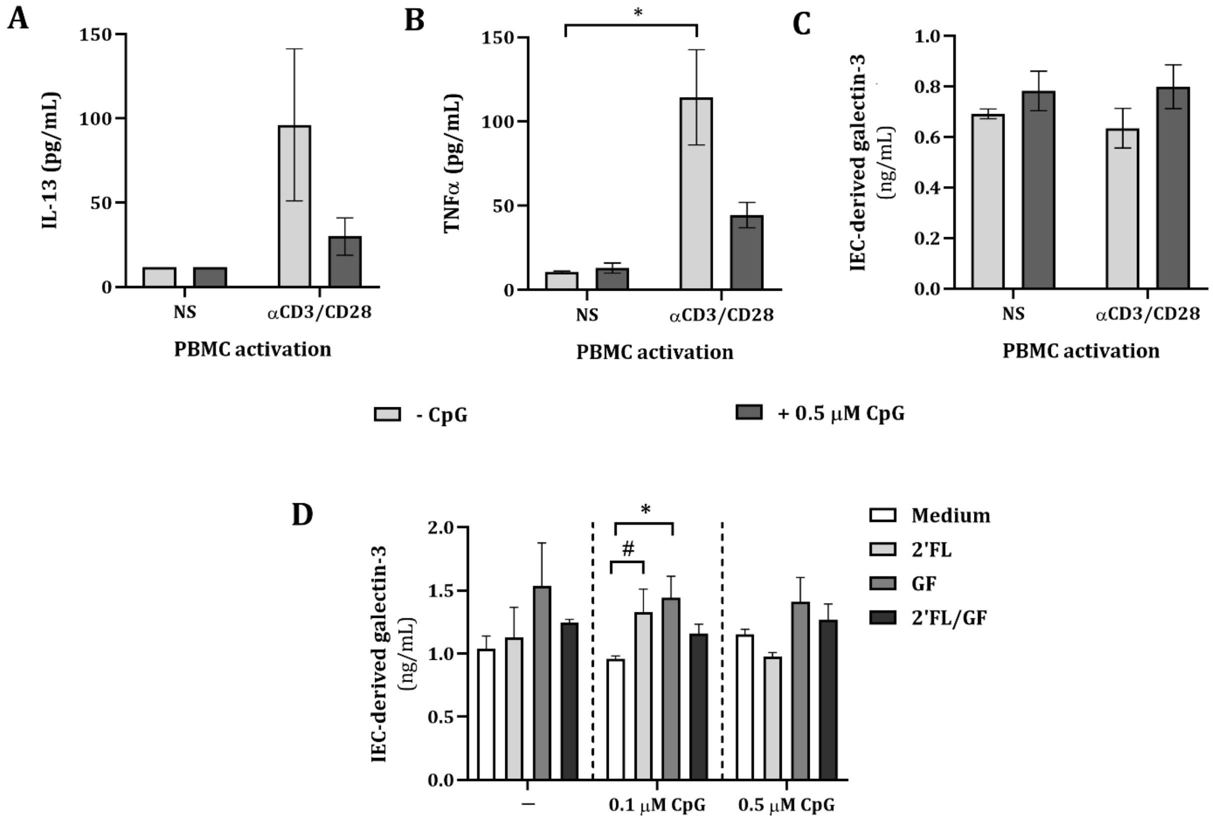

Supplementary figure 3. **Dose-response effects and correlations in IEC/PBMC co-culture**. IEC (HT-29 cell line) were stimulated with 2'FL, GF or 1:1 mixture of 2'FL and GF (0.25-1% *w/v*) in combination with CpG (0.5 μM) and basolaterally exposed to αCD3/CD28-activated PBMC for 24 h. After incubation, galectin-9 (**A**), IFNγ (**C**), IL-10 (**D**), IL-13 (**E**) and TNFα (**F**) were measured. Additionally, after IEC/PBMC co-culture, HT-29 IEC were washed and incubated in fresh medium for additional 24 h after which IEC-derived galectin-9 (**B**) was measured. The correlations between IEC-derived galectin-9 and the cytokines in IEC/PBMC co-culture are also shown (**G**). Data are represented as mean ± SEM of *n* = 7 (A, B, C, D, E, F) or *n* = 8 (D) independent PBMC donors (# $p < 0.1$, * $p < 0.05$, ** $p < 0.01$, *** $p < 0.001$).

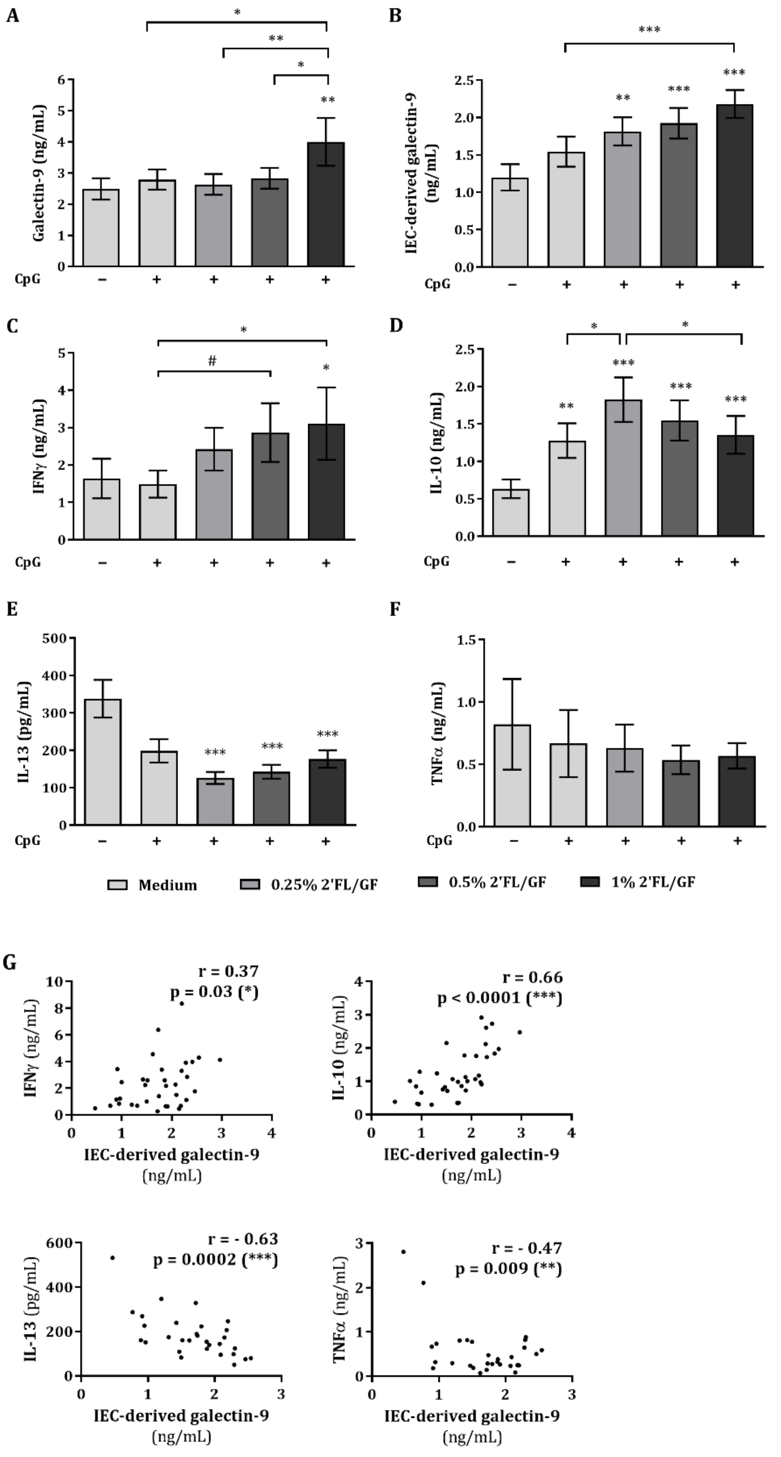

Supplementary figure 4. **Epithelial-derived galectin-3 and -4 secretion in IEC/PBMC co-culture.** After HT-29/PBMC co-culture, IEC were washed and incubated in fresh medium for additional 24h (total 48h; 24h in IEC/PBMC co-culture and additional 24h of IEC culture in fresh medium), after which basolateral supernatant was collected and epithelial-derived galectin-3 (**A**) and -4 (**B**) were measured. Data are represented as mean ± SEM of *n* = 6 (A) or *n* = 8 (B) independent PBMC donors (# $p < 0.1$, * $p < 0.05$, ** $p < 0.01$, *** $p < 0.001$).

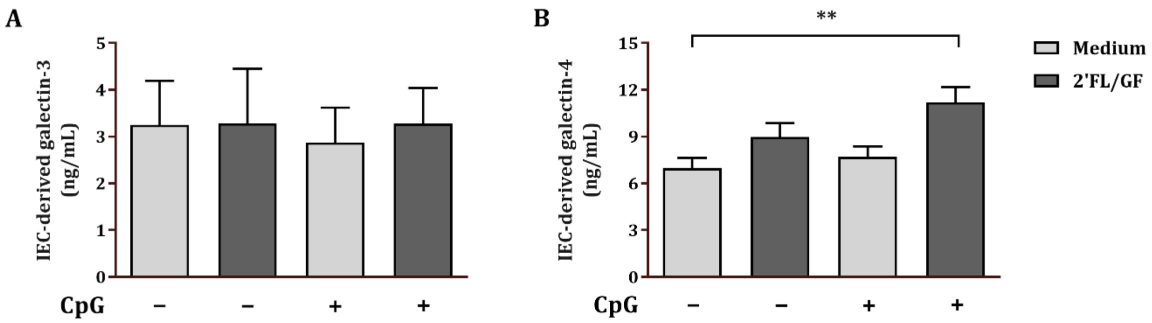

Supplementary figure 5. Correlation plots epithelial-derived galectins and cytokines in HT-29/PBMC co-culture.

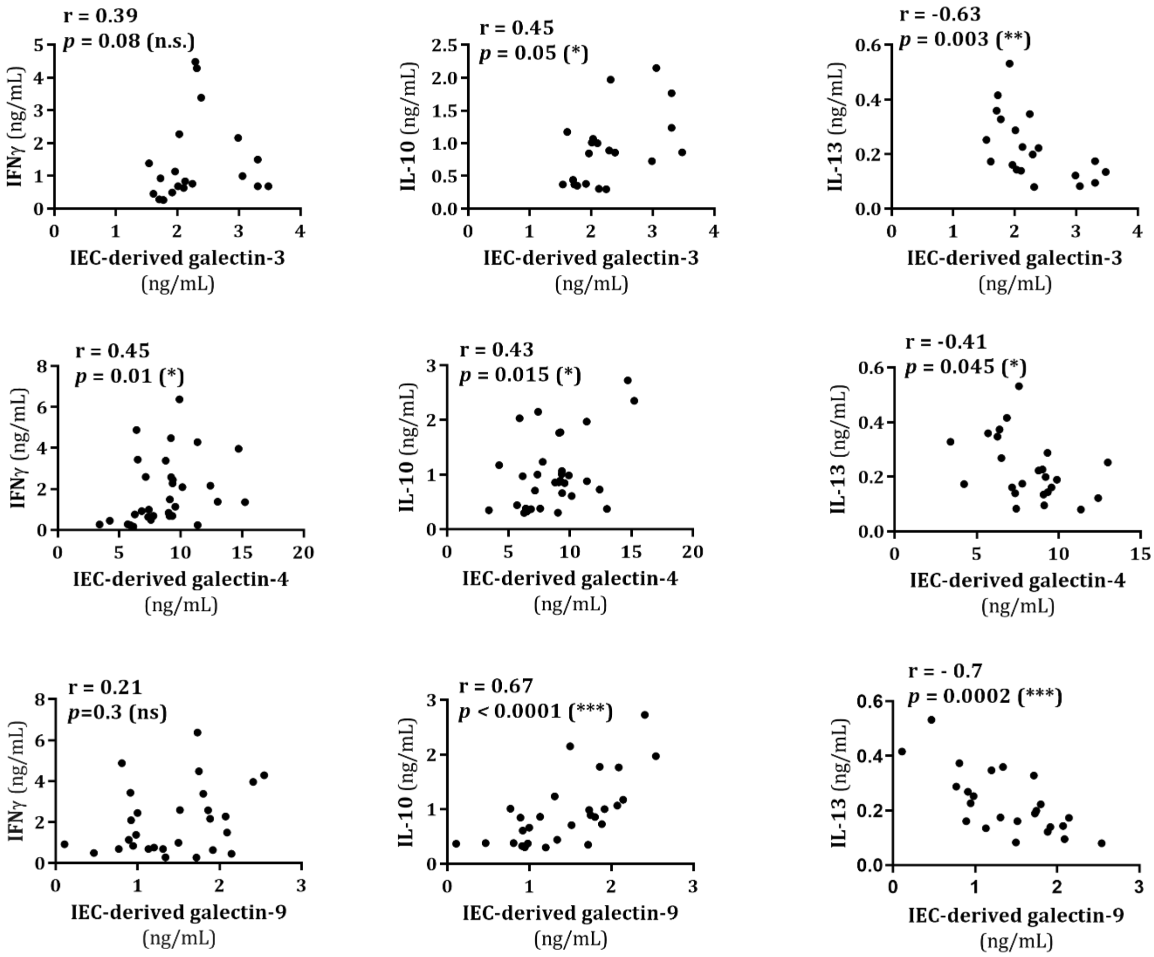

Supplement: Supplementary file 1 [file DataSheet_1.pdf]
